# The association between weekly hours of physical activity and mental health: A three-year follow-up study of 15–16-year-old students in the city of Oslo, Norway

**DOI:** 10.1186/1471-2458-7-155

**Published:** 2007-07-12

**Authors:** Aase Sagatun, Anne Johanne Søgaard, Espen Bjertness, Randi Selmer, Sonja Heyerdahl

**Affiliations:** 1 Research Department, Centre for Child and Adolescent Mental Health, Eastern and Southern Norway, PB 4623 Nydalen, 0405 Oslo, Norway; 2 Division of Epidemiology, Norwegian Institute of Public Health, PB 4404 Nydalen, 0403 Oslo, Norway; 3 Section for Preventive Medicine and Epidemiology, Institute of General Practice and Community Medicine, Faculty of Medicine, University of Oslo, PB 1130 Blindern, 0318 Oslo, Norway

## Abstract

**Background:**

Mental health problems are a worldwide public health burden. The literature concerning the mental health benefits from physical activity among adults has grown. Adolescents are less studied, and especially longitudinal studies are lacking. This paper investigates the associations between weekly hours of physical activity at age 15–16 and mental health three years later.

**Methods:**

Longitudinal self-reported health survey. The baseline study consisted of participants from the youth section of the Oslo Health Study, carried out in schools in 2000–2001 (*n *= 3811). The follow-up in 2003–2004 was conducted partly at school and partly through mail. A total of 2489 (1112 boys and 1377 girls) participated in the follow-up. Mental health was measured by the Strengths and Difficulties Questionnaire with an impact supplement. Physical activity was measured by a question on weekly hours of physical activity outside of school, defined as exertion 'to an extent that made you sweat and/or out of breath'. Adjustments were made for well-documented confounders and mental health at baseline.

**Results:**

In boys, the number of hours spent on physical activity per week at age 15–16 was negatively associated with emotional symptoms [B (95%CI) = -0.09 (-0.15, -0.03)] and peer problems [B (95%CI) = -0.08 (-0.14, -0.03)] at age 18–19 after adjustments. In girls, there were no significant differences in SDQ subscales at age 18–19 according to weekly hours of physical activity at age 15–16 after adjustments. Boys and girls with five to seven hours of physical activity per week at age 15–16 had the lowest mean scores for total difficulties and the lowest percentage with high impact score at age 18–19, but the differences were not statistically significant after adjustments.

**Conclusion:**

Weekly hours of physical activity at age 15–16 years was weakly associated with mental health at three-year follow-up in boys. Results encourage a search for further knowledge about physical activity as a possible protective factor in relation to mental health problems in adolescence.

## Background

Mental health problems are a worldwide public health burden. They decrease the quality of life and substantially increase health care costs. At any given time, one in eight children have an impairing psychiatric disorder [1], and considerably more adolescents report psychiatric symptoms [2]. The fact that symptoms of mental distress are frequent and cause significant impairment underlines the importance of knowledge about risk and protective factors in relation to mental health problems.

The documented benefits from regular physical activity for psychological well being in adults include improved mood states, enhanced self-perception and self-esteem [3]. Further, exercise has been recommended as a tool in therapy for mild to moderate depression and anxiety [3,4]. Several mechanisms have been hypothesized. Biochemical mechanisms which include release of endorphins, and increased serotonin and norepinephrine synthesis have been suggested, but methodological problems have prevented the link to mental health effects in humans [3,5,6]. Another hypothesis is that exercise reduces emotional strain and serves as a buffer against stressful events. In addition, participation in regular physical exercise programs may convey a sense of mastery and increased self-esteem [7-9]. Participation in sport and exercise groups may also provide social interaction and promote social support. Most studies reporting mental health effects of physical activity deal with relationships between physical activity and mental health within short time frames [3,4]. Little is known about the long-term effects of physical activity on the risk of developing mental health problems [4]. The small known number of prospective studies of adults concludes that inactivity is an independent predictor of depressive disorder and that physical activity protects against depression [10-15]. One study of women reports a negative association between athletic activity in college and depression 15 years later [15]. Others could not find evidence that exercise reduces the risk of depression [16] or anxiety [10,13]. Some population-based studies focus on the relation between physical activity and mental health in adolescents, but most of them are cross sectional [17-21]. The only, to our knowledge, population-based longitudinal study in young teens (7th to 8th grade) reported an association between reduced physical activity and increased depressive symptoms [22]. Thus, knowledge of the association between physical activity and various dimensions of mental health in a longitudinal perspective are lacking. In a review of the dose-response effects of physical activity on depression and anxiety, the authors noted the lack of studies focusing on frequency or duration of physical activity and symptoms of mental health problems [23]. Although both physical activity and mental health problems in adolescence differ by gender, results are rarely reported separately for boys and girls. Thus, little is known of the relationships between different amounts of physical activity in mid-teens and various mental health outcomes over time, and whether the associations differ by gender.

The aim of the present study was to investigate how numbers of weekly hours of physical activity in boys and girls at age 15–16 are associated with mental health three years later.

## Methods

### Baseline study

All 10th graders in Oslo during the school years 1999–2000 and 2000–2001 were invited to enter the youth component of the Oslo Health Study, a questionnaire study conducted in schools. All parents received written information and the students completed a consent form before participation. The students completed the two questionnaires during two school classes. A project assistant was present in the classroom to inform the students about the survey and to administer the questionnaires. Questionnaires were left at school to be completed by students not present on the day of the survey. Those who did not respond were sent a copy by mail to their home address, together with a prestamped, return envelope. A more detailed description has been published elsewhere [24]. From the total population of 15–16-year-olds, 7343 (88%) participated. Those participating in 2000–2001 (*n *= 3811) constituted the baseline of our longitudinal study and were invited again in 2004.

### Follow-up study

The follow-up study was carried out partly as a school-based survey and partly through mail. The study is described more thoroughly elsewhere [25]. The procedure of the school-based part of the study was similar to the baseline. All the 32 secondary high schools in Oslo took part and the final year students filled out one questionnaire during one school class.

The participants in the baseline study (2000–2001) who were not enrolled in the final year of secondary high school in Oslo and who had consented to participate in a follow-up were invited by mail to participate. The invitation included an invitation letter, an information brochure, a consent form, the questionnaire and a prestamped return envelope. Two reminders were sent to those who did not respond.

### Study population

From the participants in the baseline study (3811), 2489 (1112 boys and 1377 girls) participated in the follow-up with consent to link data from the two surveys. Students reporting motor disabilities at baseline were excluded (*n *= 56). Only adolescents who had participated in both surveys were included in the analysis (*n *= 2433) [see Additional file 1]. Those who did not respond in the follow-up were characterized at baseline by higher mean (SD) SDQ total (10.8 (4.9) vs. 9.6 (4.8), *p *< 0.001) and lower physical activity levels (*p *< 0.001). The association between SDQ total score and physical activity at baseline among the responders and non-responders was similar (*p*^(interaction) ^= 0.725).

### Measures

#### Mental health

We used the self-report version of the Strengths and Difficulties Questionnaire (SDQ) [26]. The SDQ has been used in large number of studies during the last ten years, including the British Child and Adolescent Mental Health Survey [27], The US National Health Interview Survey [28], and several large Norwegian epidemiological studies [29]. The SDQ is a 25-item screening questionnaire with five scales, each consisting of five items, generating scores for emotional symptoms, conduct problems, hyperactivity-inattention, peer problems, and prosocial behaviour. Each item can be answered with 'not true' (0), 'somewhat true' (1) or 'certainly true' (2). The first four problem scales are summed to generate a total difficulties score. SDQ is designed and validated for youngsters (11–16 years), but SDQ has also been used for older youths [29]. In the follow-up questionnaire minor linguistic changes were made in accordance with the approved Norwegian translation. The internal consistency (Cronbach's alpha) of the various SDQ scales at baseline and follow-up were: 0.73, 0.77 for the total difficulties score; 0.70, 0.73 for emotional symptoms; 0.47, 0.38 for conduct problems; 0.54, 0.65 for hyperactivity-inattention; 0.53, 0.57 for peer problems; and 0.64, 0.61 for prosocial behaviour. In accordance with other studies, the Cronbach's alpha values were low for some of the subscales [30], particularly for conduct problems [31]. The problem scales are based on current nosological concepts [30]. Conduct problem items cover selected essential criteria for oppositional defiant disorder and conduct disorder [32]. A large validation study found that a high score on the self-report conduct problems was associated with an odds ratio of 7.1 for having conduct or oppositional-defiant disorder [30]. The self-report version of SDQ has also shown satisfactory discrimination between community and clinical samples [33].

Many young people with high psychiatric symptoms score in epidemiological studies, are not significantly socially impaired by their symptoms [34]. To get a better indication of influence of the symptoms an impact supplement has been made [35]. This supplement was included in our follow-up survey and starts with a question on whether the respondent thinks he or she has a problem. If so, further enquiries are made about chronicity, overall distress, social impairment and burden to the environment. The impact questions have four response categories: no (0), little (0), quite a lot (1), a great deal (2). The items concerning overall distress and social impairment related to family, friends, learning situation and leisure activities generate a total impact score, ranging between 0 and 10. A validation study finds that the impact scores discriminate between a clinical and a community sample, and that impact scores were better than symptoms scores at discriminating between the two samples [35]. Goodman defines a score of two or more as 'abnormal' or 'caseness' and a score of one as borderline [35]. The variable is dichotomized into high (≥1) and low (<1) scores when conducting logistic regression.

#### Weekly hours of physical activity in leisure time

Participants were asked how many hours per week they spend on physical activity 'to an extent that make you sweat and/or out of breath'; 0, 1–2, 3–4, 5–7, 8–10, or 11 hours or more per week. When studying the relationship between physical activity and mental health by variance analysis and logistic regression, physical activity was recoded into four groups (0, 1–4, 5–7 and ≥8 hours per week). The six original categories were included as an ordinal variable coded 1,2,..6 in multiple linear regression.

#### Confounding factors

Adjustments were done for well-documented confounding factors: ethnic background, family economy, smoking, and use of alcohol [36-39]. Of the study population, 20% reported an ethnic minority background, defined as those having both parents born in a country other than Norway [40]. The family economic status was characterized as 'very bad/bad', 'good' or 'very good' based on a question comparing the family economy with other families in Norway. Alcohol consumption was measured by asking how often in course of the past year the person had drunk alcohol, and coded into six categories (Table 1). Smoking habits were categorized into 'never/quit', 'once in a while' and 'daily'.

**Table 1:** Baseline characteristics of participants in the youth part of the Oslo Health Study (2000–2001) who participated in the follow-up in 2004.

**Characteristics at age 15–16**	**Boys**		**Girls**		
			
	*n *= 1085*	%	*n *= 1348*	%	*p, gender*
**Invitation group**					*0.489*
In school	*913*	84.1	*1148*	85.2	
By mail	*172*	15.9	*200*	14.8	
**Ethnic background **					*0.099*
Ethnic Norwegian	*879*	81.5	*1057*	78.8	
Ethnic minority	*200*	18.5	*285*	21.2	
**Family economic status**					*0.047*
Very bad/Bad	*285*	26.6	*399*	30.2	
Good	*634*	59.2	*771*	58.3	
Very good	*152*	14.2	*152*	11.5	
**Alcohol use last year**					*0.094*
2–3 times per week	*42*	3.9	*51*	3.8	
Once per week	*137*	12.9	*204*	15.4	
2–3 times per month	*233*	21.9	*245*	18.5	
Once per month	*94*	8.8	*128*	9.6	
A few times	*247*	23.2	*342*	25.8	
Not at all	*313*	29.4	*357*	26.9	
**Smoking**					*<0.001*
No	*844*	78.2	*939*	70.0	
Once in a while	*152*	14.1	*232*	17.3	
Yes, daily	*83*	7.7	*171*	12.7	
**Physical activity**					*<0.001*
0 hours per week	*82*	7.6	*143*	10.6	
1–2 hours per week	*202*	18.8	*418*	31.0	
3–4 hours per week	*223*	20.7	*361*	26.8	
5–7 hours per week	*263*	24.4	*234*	17.4	
8–10 hours per week	*187*	17.4	*96*	7.1	
11 hours or more per week	*120*	11.1	*60*	4.5	

*The numbers who answered the different questions differ somewhat.

Because the study was conducted partly in school and partly by mail, we created a variable, 'Invitation group', categorizing mail or school participation.

#### Ethics

Both protocols were evaluated by the Regional Committee for Medical Research Ethics and were approved by the Norwegian Data Inspectorate. The baseline study, and the part of the follow-up study carried out in the schools, received approval from the school authorities in Oslo.

### Statistical methods

In addition to descriptive statistics, paired and un-paired t-testes, we used the GLM Repeated Measure to test if change in SDQ in the follow-up period differed between boys and girls. One-way ANCOVA between-groups analysis of covariance was used to test if the SDQ subscales in the follow-up varied by weekly hours of physical activity (0, 1–4, 5–7 or ≥8) at baseline. Invitation group, ethnic background, family economy, smoking and alcohol use, and the respective SDQ subscale at baseline were collectively entered as covariates. Results are given as adjusted means with 95% confidence intervals (95% CI). To test the linearity of the associations described in the ANOVA/ANCOVA we performed multiple linear regression. To test a nonlinear association between physical activity and mental health, physical activity squared [(physical activity)^2^] was included in the model. Logistic regression was conducted to study how those with high total impact score at follow-up varied between the four groups of physical activity at baseline. All analyses were done separately for boys and girls. Calculations were performed in SPSS 13.

## Results

Baseline characteristics are presented in Table 1. There was no gender difference in ethnic background, invitation group and alcohol consumption. Girls perceived their family economic status to be slightly worse than did boys. Girls were more often daily or 'once in a while' smokers than boys, and boys spent more hours per week on physical activity compared with girls (Table 1).

At baseline, girls reported more overall mental health difficulties (SDQ total difficulties), emotional symptoms and hyperactivity-inattention problems than boys. Girls also reported more prosocial behaviour than boys, whereas boys reported more conduct problems and peer problems than girls (Table 2). During the follow-up period, the difference in overall mental health difficulties between the genders increased, with a reduction in SDQ total difficulties for boys and an increase for girls. Peer problems were reduced in boys and increased in girls. Emotional symptoms also increased in girls, while there was no change in boys. Both genders had an increase in hyperactivity-inattention problems and prosocial behaviour, while conduct problems decreased (Table 2).

**Table 2:** Strengths and Difficulties Questionnaire (SDQ)  mean score at age 15–16 years (baseline) and at age 18–19 years (follow-up) in boys and girls in Oslo (2001–2004).

**Measure and Time Point**	**Boys**				**Girls**				
			
	*n*	mean	SD	p time**	*n*	mean	SD	p time**	p, gender***
SDQ -Emotional Symptoms				*0.183*				*0.021*	
*baseline*	*1074*	1.64	1.69		*1340*	3.38	2.27		*<0.001*
*follow up*	*1082*	1.73	1.69		*1343*	3.52	2.41		*<0.001*
SDQ – Conduct Problems*				*<0.001*				*<0.001*	
*baseline*	*1076*	2.07	1.67		*1344*	1.88	1.37		*0.002*
*follow up*	*1083*	1.67	1.39		*1343*	1.69	1.22		*0.684*
SDQ – Hyperactivity-Inattention				*0.038*				*<0.001*	
*baseline*	*1074*	3.22	1.93		*1339*	3.62	1.90		*<0.001*
*follow up*	*1083*	3.35	2.07		*1344*	3.88	2.11		*<0.001*
SDQ – Peer Problems*				*0.009*				*0.008*	
*baseline*	*1074*	1.61	1.56		*1340*	1.39	1.39		*<0.001*
*follow up*	*1083*	1.50	1.54		*1344*	1.51	1.53		*0.874*
SDQ -Prosocial Behavior*				*<0.001*				*<0.001*	
*baseline*	*1077*	6.95	1.87		*1344*	7.98	1.55		*<0.001*
*follow up*	*1083*	7.69	1.71		*1342*	8.38	1.40		*<0.001*
SDQ -Total Difficulties*				*0.019*				*0.011*	
*baseline*	*1072*	8.53	4.63		*1339*	10.27	4.64		*<0.001*
*follow up*	*1082*	8.25	4.51		*1342*	10.60	5.12		*<0.001*

*General Linear Model Repeated Measure showed a statistical interaction between time and gender (p < 0.001)

**Paired t-test

***Un-paired t-test

### Mental health at age 18–19 years according to physical activity at age 15–16 years

Emotional symptoms and peer problems at age 18–19 were inversely associated with physical activity at age 15–16 in both genders, whereas prosocial behaviour was positively associated only in boys (p < 0.05 for all these associations in linear regression analyses) (Table 3, crude analysis). Boys and girls who were physically active 5–7 hours per week at age 15–16 had the lowest total problem score at follow up [see Additional file 2]. The U-shaped association was statistically significant in regression models, with (physical activity)^2 ^included, for both boys (*p *= 0.003) and girls (*p *= 0.014). When adjusting for invitation group, ethnic background, family economic status, smoking, use of alcohol and the respective SDQ score at baseline, the U-shaped trend between SDQ total and physical activity was no longer statistically significant for neither boys (*p *= 0.14) nor girls (*p *= 0.31). Also the differences in the SDQ subscales according to physical activity decreased after adjustments (Table 3, adjusted model). In girls, there were no significant differences between categories of physical activity at baseline for any SDQ subscale at follow up after adjustments (Table 3, adjusted model), whereas emotional problems [B (95%CI) = -0.09 (-0.15, -0.03)] and peer problems [B (95%CI) = -0.08 (-0.14, -0.03)] decreased with increasing amount of physical activity in boys. In boys, prosocial behaviour varied significantly between the groups of physical activity (Table 3, adjusted model) but there was no significant linear trend [B (95%CI) = 0.05 (-0.02, 0.12)].

**Table 3:** Strengths and Difficulties Questionnaire (SDQ) according to weekly hours of physical activity. SDQ (mean scores) at age 18–19 years according to hours of physical activity per week at age 15–16 years, in boys and girls – unadjusted (crude) and adjusted for confounders and mental health at baseline.

**Physical activity per week** ** (age 15–16)**	**Strengths and Difficulties Questionnaire (age 18–19)**																			
	**Boys**										**Girls**									
		
	Crude					Adjusted*					Crude					Adjusted*				
				
	*n*	mean	95%CI	F	*p*	*n*	mean	95%CI	F	*p*	*n*	mean	95%CI	F	*p*	*n*	mean	95%CI	F	*p*
**SDQ – Emotional Symptoms**				5.91	*0.001*				3.28	*0.020*				6.14	*<0.001*				0.08	*0.971*
*0 hours*	*80*	2.00	(1.60–2.40)			*73*	1.80	(1.46–2.14)			*141*	4.05	(3.64–4.46)			*131*	3.54	(3.19–3.89)		
*1–4 hours*	*424*	1.96	(1.78–2.13)			*410*	1.86	(1.72–2.01)			*777*	3.59	(3.42–3.76)			*750*	3.48	(3.34–3.62)		
*5–7 hours*	*263*	1.51	(1.32–1.69)			*254*	1.56	(1.38–1.75)			*233*	3.09	(2.80–3.37)			*227*	3.46	(3.20–3.73)		
*8 hours or more*	*307*	1.55	(1.38–1.73)			*299*	1.56	(1.40–1.73)			*156*	3.18	(2.80–3.56)			*151*	3.54	(3.22–3.87)		
**SDQ – Conduct Problems**				2.90	*0.087*				1.28	*0.280*				1.69	*0.168*				1.09	*0.353*
*0 hours*	*81*	1.62	(1.35–1.89)			*74*	1.57	(1.30–1.85)			*141*	1.89	(1.69–2.08)			*131*	1.69	(1.50–1.88)		
*1–4 hours*	*424*	1.71	(1.57–1.84)			*412*	1.71	(1.60–1.83)			*777*	1.64	(1.56–1.73)			*753*	1.63	(1.55–1.71)		
*5–7 hours*	*263*	1.51	(1.34–1.67)			*253*	1.58	(1.43–1.73)			*233*	1.65	(1.51–1.79)			*227*	1.70	(1.56–1.84)		
*8 hours or more*	*307*	1.79	(1.64–1.95)			*300*	1.75	(1.62–1.89			*156*	1.72	(1.53–1.92)			*151*	1.80	(1.63–1.97)		
**SDQ – Hyperactivity- Inattention**				1.14	*0.331*				0.92	*0.431*				1.14	*0.331*				0.47	*0.700*
*0 hours*	*81*	3.48	(3.00–3.96)			*74*	3.31	(2.89–3.73)			*141*	4.08	(3.75–4.41)			*131*	3.95	(3.63–4.27)		
*1–4 hours*	*424*	3.23	(3.04–3.42)			*410*	3.27	(3.09–3.45)			*778*	3.78	(3.63–3.92)			*751*	3.80	(3.67–3.93)		
*5–7 hours*	*263*	3.32	(3.08–3.56)			*254*	3.34	(3.11–3.57)			*233*	3.89	3.61–4.17)			*227*	3.90	(3.66–4.14)		
*8 hours or more*	*307*	3.50	(3.25–3.74)			*299*	3.50	(3.29–3.71)			*156*	3.99	(3.65–4.33)			*151*	3.93	(3.63–4.22)		
**SDQ – Peer Problems**				13.3	*<0.001*				2.57	*0.053*				4.41	*0.004*				2.10	*0.098*
*0 hours*	*81*	2.20	(1.83–2.56)			*74*	1.79	(1.48–2.10)			*141*	1.90	(1.62–2.18)			*131*	1.74	(1.50–1.98)		
*1–4 hours*	*424*	1.69	(1.54–1.84)			*409*	1.52	(1.39–1.65)			*777*	1.49	(1.38–1.60)			*750*	1.43	(1.34–1.53)		
*5–7 hours*	*263*	1.21	(1.04–1.38)			*254*	1.40	(1.23–1.56)			*234*	1.32	(1.13–1.50)			*227*	1.47	(1.29–1.65)		
*8 hours or more*	*307*	1.28	(1.11–1.45)			*300*	1.35	(1.20–1.50)			*156*	1.49	(1.24–1.73)			*151*	1.57	(1.35–1.79)		
SDQ – Prosocial Behavior**				5.00	*0.002*				4.44	*0.004*				0.96	*0.411*				0.79	*0.490*
*0 hours*	*81*	7.14	(6.72–7.55)			*74*	7.40	(7.04–7.75)			*141*	8.43	(8.22–8.65)			*131*	8.50	(8.28–8.72)		
*1–4 hours*	*424*	7.77	(7.61–7.94)			*412*	7.75	(7.60–7.90)			*778*	8.33	(8.23–8.44)			*754*	8.34	(8.25–8.43)		
*5–7 hours*	*263*	7.53	(7.32–7.73)			*254*	7.44	(7.25–7.63)			*233*	8.39	(8.21–8.57)			*227*	8.42	(8.25–8.58)		
*8 hours or more*	*307*	7.86	(7.67–8.04)			*300*	7.86	(7.68–8.04)			*156*	8.53	(8.32–8.74)			*151*	8.42	(8.22–8.63)		
**SDQ – Total Difficulties**				4.31	*0.005*				0.67	*0.579*				4.63	*0.003*				1.03	*0.377*
*0 hours*	*80*	9.24	(8.22–10.25)			*73*	8.32	(7.43–9.21)			*141*	11.91	(11.07–12.76)			*131*	10.88	(10.16–11.60)		
*1–4 hours*	*424*	8.58	(8.14–9.02)			*409*	8.35	(7.98–8.73)			*776*	10.51	(10.15–10.87)			*749*	10.35	(10.06–10.65)		
*5–7 hours*	*263*	7.54	(7.01–8.07)			*253*	7.93	(7.45–8.40)			*233*	9.94	(9.31–10.56)			*227*	10.56	(10.02–11.10)		
*8 hours or more*	*307*	8.13	(7.62–8.63)			*299*	8.15	(7.72–8.59)			*156*	10.38	(9.57–11.20)			*151*	10.83	(10.17–11.49)		

*Adjusted for: invitation group, ethnicity, family economy, smoke, use of alcohol, and the respective SDQ subscore at baseline.

**High values are positive

Boys and girls who were physically active 5–7 hours per week at age 15–16 years had the lowest risk of reporting distress and social impairment (total impact score ≥1) three years later (Table 4, crude model). However after adjusting for possible confounders and mental health at baseline the associations were no longer statistically significant (Table 4, adjusted model).

**Table 4:** Total Impact score according to weekly hours of physical activity. Percentage (p%) with high score*  and odds ratio (OR) for high score* at age 18–19 years according to hours of physical activity per week at age 15–16 years, in boys and girls–unadjusted (crude) and adjusted for confounders and mental health at baseline.

**Physical activity per week** ** (age 15–16)**	**Boys**						**Girls**					
		
			Crude		Adjusted**				Crude		Adjusted**	
				
	*n*	p%***	OR	95%Cl	OR	95%Cl	*n*	p%***	OR	95%Cl	OR	95%Cl
*0 hours*	*79*	22.8	ref.		ref.		*139*	36.7	ref.		ref.	
*1–4 hours*	*423*	17.3	0.71	0.39 – 1.27	0.79	0.42 – 1.49	*775*	28.8	0.67	0.46 – 0.98	0.82	0.53 – 1.24
*5–7 hours*	*262*	10.3	0.39	0.20 – 0.75	0.51	0.25 – 1.04	*232*	24.6	0.56	0.36 – 0.89	0.76	0.45 – 1.27
*8 or more hours*	*304*	17.1	0.70	0.38 – 1.28	0.84	0.44 – 1.64	*155*	29.7	0.73	0.45 – 1.19	1.05	0.61 – 1.81

*High total impact score ≥1

**Invitation group, Ethnicity, Family economy, smoking and alcohol habit, SDQ total at baseline

***Percent of n with high total impact score

## Discussion

In boys, weekly hours of physical activity at age 15–16 years were inversely associated with emotional symptoms and peer problems at age 18–19 years after adjustment for confounders and mental health at baseline. In boys also prosocial behaviour varied with physical activity after adjustments, but there was no linear trend. In girls there was no independent effect of physical activity at age 15–16 on mental health at age 18–19 after adjustments. Participants reporting five to seven hours of physical activity per week at age 15–16 had the lowest mean score for SDQ total difficulties and the lowest percentage with high impact score at follow up, but the differences were not statistically significant after adjustments.

### Methodological strengths and limitations

Our longitudinal study extends beyond previous reports of population-based cross-sectional associations between physical activity and depression [19], emotional well-being [20] and social problems [17,18] when studying physical activity at age 15–16 years and different aspects of mental health three years later. We are still not able to infer the direction of causality from our analysis. There could be common underlying factors influencing both physical activity and mental health at both time points, or these two factors could be in a continuous circular relationship.

In the baseline study, all 10th graders in Oslo were invited to participate in the school year 2000–2001. The high participation rate implies that the baseline cohort is representative for 10^th ^graders in Oslo. In the follow-up, those not participating were less physically active and reported more symptoms of mental health problems at baseline than those who participated. However, the association at baseline between SDQ total problem score and physical activity was similar for the non-responders and those participating in the follow-up. Thus, we believe it is unlikely that those lost to follow-up would appreciably influence the associations at follow-up.

When measuring mental health among children and adolescents it is preferable to use several informants (also parents and teachers). Self-reports are generally less strongly associated with psychiatric disorder than parent reports [30]. In our follow-up study the participants were 18 years, and parent reports could not be included. The fact that this is a self-report study, with low internal consistency for some of the subscales, should be kept in mind when interpreting the results. However, low internal consistency may have led to an underestimation of associations.

Measuring physical activity by questionnaire is also associated with difficulties [41]. We have used a single item measure for this variable, something that has to be taken into consideration. A measure of weekly hours of physical activity however, has shown the ability to discriminate between levels of aerobic fitness in youth [42]. It seems reasonable to assume that the question captures different levels of physical activity, but how accurate the adolescents report hours peer week is unknown.

Within the limitation of the study design the results reveal an interesting association between physical activity and mental health. Although the associations were weak, physical activity was a positive factor for some dimensions of mental health problems in boys. In the population strategy of prevention: 'even a small shift in the distributions may have a large effect on the number of individuals falling into the high vulnerable tail of the distribution' [43].

### Mental health at age 18–19 years according to physical activity at age 15–16 years

We found a more consistent association between weekly physical activity and mental health in boys than in girls, and the associations were connected to emotional symptoms, peer problems and prosocial behaviour. Similar overall findings have been reported from cross-sectional studies of adolescents [17-19], but not by gender. One such study of adolescents in grades 7–13 found a positive association between vigorous physical activity and social functioning, but no association for depression/anxiety when adjusted for age, gender, and SES [17]. Kirkcaldy et al reported that adolescents who engaged regularly in physical activity display much less inhibition in social behaviour than their less active counterparts [18]. A third cross-sectional study reported that adolescents who did not exercise, or exercised infrequently, scored higher on psychological discomfort measured by loneliness, shyness and hopelessness than did adolescents who were more frequent exercisers [19]. One explanation for all these findings may be that physical activity and the sports arena represent an increased opportunity for social interaction and development of social skills. Adolescents who join a sports club show lower anxiety and depression scores than those pursuing individual sports [44]. Boys are more often than girls doing sport in a club [45]. If physical activity promotes social development, it is likely that this is a more permanent influence – and may be possible to detect as a long-term effect. Cardon et al found in a study among 1124 children aged 10 and 11 that boys felt more social support from family and friends to be active than girls [46]. Boys also perceived more benefits from physical activity than girls in regard to being together with friends/meet people, have fun and being admired by others. The difference in peer problems and prosocial behavior according to physical activity may also be due to individuals with low social competence ceasing physical activity or participating in sports at a young age. Adolescents who possess good sport or physical activity skills may also be more socially attractive than less talented peers. Different patterns in type of physical activity, and changes in weekly hours over the period under study may also contribute to the gender difference.

Participants reporting 5–7 hours of physical activity per week at age 15–16 had *both *the lowest SDQ total problem score and the lowest percentage with SDQ total impact score at follow-up, but the differences became insignificant after adjustments. Cross sectional studies have however reported similar U-shaped associations [19,21,47]. One suggested reason for the non-linear association involves the detrimental effect of overtraining in athletes [47]. Unger (1997), who studied participation in sport team and suicidal behaviour, revealed that girls who exercised 6–7 days per week and did not participate in team sports, were at the greatest risk of suicidal behaviour [21]. The author of this study suggests that these results may be due to a widespread perception of overweight and negative body image among adolescent girls who then exercise to lose weight. More research in general populations is needed on effects of high volume physical activity/exercise and mental health among adolescents.

## Conclusion

Our findings indicate that physical activity at age 15–16 years may influence some aspects of mental health three years later in boys, but not in girls. Our study underlines the need to perform longitudinal studies with different aspect of mental health as outcome, conducting separate analyses by gender, and also consider the amount of physical activity when studying the relation between physical activity and mental health in adolescence. The results encourage subsequent cohort studies to focus on different types of physical activity (competitive vs. recreational, team vs. individual) and mental health follow up.

## Competing interests

The author(s) declare that they have no competing interests.

## Authors' contributions

AaS was active in the planning of the follow-up and coordinated the practical part of the study, did the conception and design, analysed and interpreted the data and drafted the manuscript. AJS was project manager of the baseline study, participated in planning of the follow-up study, was involved in the conception and design of the project, discussed the analysis and interpretation of the data, and reviewed the article critically. EB was project manager of the follow-up study, involved in the conception and design of the project, discussed the analysis and interpretation of the data, and reviewed the article critically. RS took part in collecting of the baseline data, contributed particularly in the statistical analyses and interpretation of the data, and reviewed the article critically. SH was involved in the conception and design of the project, discussed the analysis and interpretation of the data and reviewed the article critically.

## Pre-publication history

The pre-publication history for this paper can be accessed here:



## Supplementary Material

Additional file 1 Flowchart of the study and the participants at follow-up. Click here for file

Additional file 2 Strengths and Difficulties Questionnaire Total difficulties score (follow up) according to weekly hours of physical activity (baseline) in boys and girls. Click here for file

## References

[B1] Costello EJ, Egger H, Angold A (2005). 10-year research update review: the epidemiology of child and adolescent psychiatric disorders: I. Methods and public health burden. J Am Acad Child Adolesc Psychiatry.

[B2] Roberts RE, Attkisson CC, Rosenblatt A (1998). Prevalence of psychopathology among children and adolescents. Am J Psychiatry.

[B3] Fox KR (1999). The influence of physical activity on mental well-being. Public Health Nutr.

[B4] Paluska SA, Schwenk TL (2000). Physical activity and mental health. Current concepts. Sports Med.

[B5] Morgan WPe (1997). Physical activity and mental health.

[B6] Haskell WL, Bouchard C, Blair SN, Eds (2007). Physical activity and health.

[B7] Ekeland E, Heian F, Hagen KB, Abbott J, Nordheim L (2004). Exercise to improve self-esteem in children and young people. The Cochrane Database of Systematic Reviews : Reviews 2004 Issue 1.

[B8] Spence JC, McGannon KR, Poon P (2005). The Effect of Exercise on Global Self-Esteem: A Quantitative Review. JSEP.

[B9] Fox KR (2000). Self-esteem, self-perceptions and exercise. Int J Sport Psychol.

[B10] Backmand H, Kaprio J, Kujala U, Sarna S (2003). Influence of physical activity on depression and anxiety of former elite athletes. Int J Sports Med.

[B11] Camacho TC, Roberts RE, Lazarus NB, Kaplan GA, Cohen RD (1991). Physical activity and depression: evidence from the Alameda County Study. Am J Epidemiol.

[B12] Farmer ME, Locke BZ, Moscicki EK, Dannenberg AL, Larson DB, Radloff LS (1988). Physical activity and depressive symptoms: the NHANES I Epidemiologic Follow-up Study. Am J Epidemiol.

[B13] Morgan WP, Costill DL (1996). Selected psychological characteristics and health behaviors of aging marathon runners: a longitudinal study. Int J Sports Med.

[B14] Paffenbarger RS, Lee IM, Leung R (1994). Physical activity and personal characteristics associated with depression and suicide in American college men. Acta Psychiatr Scand Suppl.

[B15] Wyshak G (2001). Women's college physical activity and self-reports of physician-diagnosed depression and of current symptoms of psychiatric distress. J Womens Health Gend Based Med.

[B16] Cooper-Patrick L, Ford DE, Mead LA, Chang PP, Klag MJ (1997). Exercise and depression in midlife: a prospective study. Am J Public Health.

[B17] Allison KR, Adlaf EM, Irving HM, Hatch JL, Smith TF, Dwyer JJ, Goodman J (2005). Relationship of vigorous physical activity to psychologic distress among adolescents. J Adolesc Health.

[B18] Kirkcaldy BD, Shephard RJ, Siefen RG (2002). The relationship between physical activity and self-image and problem behaviour among adolescents. Soc Psychiatry Psychiatr Epidemiol.

[B19] Page RM, Tucker LA (1994). Psychosocial discomfort and exercise frequency: an epidemiological study of adolescents. Adolescence.

[B20] Steptoe A, Butler N (1996). Sports participation and emotional wellbeing in adolescents. Lancet.

[B21] Unger JB (1997). Physical activity, participation in team sports, and risk of suicidal behavior in adolescents. Am J Health Promot.

[B22] Motl RW, Birnbaum AS, Kubik MY, Dishman RK (2004). Naturally occurring changes in physical activity are inversely related to depressive symptoms during early adolescence. Psychosom Med.

[B23] Dunn AL, Trivedi MH, O'Neal HA (2001). Physical activity dose-response effects on outcomes of depression and anxiety. Med Sci Sports Exerc.

[B24] Søgaard A-J, Eide T Health Study (HUBRO) – The Youth part (UNGHUBRO): Methods. http://www.fhi.no/dav/831c96A203.doc.

[B25] Sagatun A, Søgaard A-J, Bjertness E (2004). Youth. Methods.

[B26] Information for researchers and professionals about the Strengths & Difficulties Questionnaires [homepage on the Internet]. youthinmind. http://www.sdqinfo.com/.

[B27] Meltzer H, Gatward R, Goodman R, Ford T (2003). Mental health of children and adolescents in Great Britain. International Review of Psychiatry.

[B28] Bourdon KH, Goodman R, Rae DS, Simpson G, Koretz DS (2005). The Strengths and Difficulties Questionnaire: U.S. normative data and psychometric properties. J Am Acad Child Adolesc Psychiatry.

[B29] Van Roy B, Groholt B, Heyerdahl S, Clench-Aas J (2006). Self-reported strengths and difficulties in a large Norwegian population 10–19 years : age and gender specific results of the extended SDQ-questionnaire. Eur Child Adolesc Psychiatry.

[B30] Goodman R (2001). Psychometric properties of the strengths and difficulties questionnaire. J Am Acad Child Adolesc Psychiatry.

[B31] Ronning JA, Handegaard BH, Sourander A, Morch WT (2004). The Strengths and Difficulties Self-Report Questionnaire as a screening instrument in Norwegian community samples. Eur Child Adolesc Psychiatry.

[B32] American Psychiatric Association (1994). Diagnostic and statistical manual of mental disorders Washington, DC.

[B33] Goodman R, Meltzer H, Bailey V (1998). The Strengths and Difficulties Questionnaire: a pilot study on the validity of the self-report version. Eur Child Adolesc Psychiatry.

[B34] Bird HR, Yager TJ, Staghezza B, Gould MS, Canino G, Rubio-Stipec M (1999). Impairment in the epidemiological measurement of childhood psychopathology in the community. J Am Acad Child Adolesc Psychiatry.

[B35] Goodman R (1999). The extended version of the Strengths and Difficulties Questionnaire as a guide to child psychiatric caseness and consequent burden. J Child Psychol Psychiatry.

[B36] Lewinsohn PM, Rohde P, Seeley JR (1998). Major depressive disorder in older adolescents: prevalence, risk factors, and clinical implications. Clin Psychol Rev.

[B37] Moore MJ, Werch CE (2005). Sport and physical activity participation and substance use among adolescents. J Adolesc Health.

[B38] Sallis JF, Prochaska JJ, Taylor WC (2000). A review of correlates of physical activity of children and adolescents. Med Sci Sports Exerc.

[B39] Schmitz KH, Lytle LA, Phillips GA, Murray DM, Birnbaum AS, Kubik MY (2002). Psychosocial correlates of physical activity and sedentary leisure habits in young adolescents: The teens eating for energy and nutrition at school study. Prev Med.

[B40] Lie B (2002). Immigration and immigrants 2002.

[B41] Shephard RJ (2003). Limits to the measurement of habitual physical activity by questionnaires. Br J Sports Med.

[B42] Booth ML, Okely AD, Chey T, Bauman A (2001). The reliability and validity of the physical activity questions in the WHO health behaviour in schoolchildren (HBSC) survey: a population study. Br J Sports Med.

[B43] Rose G (1993). The strategy of preventive medicine.

[B44] Vilhjalmsson R, Thorlindsson T (1992). The Integrative and Physiological-Effects of Sport Participation – A Study of Adolescents 14. Sociological Quarterly.

[B45] Ferron C, Narring F, Cauderay M, Michaud PA (1999). Sport activity in adolescence: associations with health perceptions and experimental behaviours. Health Educ Res.

[B46] Cardon G, Philippaerts R, Lefevre J, Matton L, Wijndaele K, Balduck AL, De Bourdeaudhuij I (2005). Physical activity levels in 10- to 11-year-olds: clustering of psychosocial correlates. Public Health Nutr.

[B47] Sanders CE, Field TM, Diego M, Kaplan M (2000). Moderate involvement in sports is related to lower depression levels among adolescents. Adolescence.

