# Supplementary figures and images for: The association between weekly hours of physical activity and mental health: A three-year follow-up study of 15–16-year-old students in the city of Oslo, Norway
Source: BMC Public Health. 2007 Jul 12;7:155. doi: 10.1186/1471-2458-7-155 (PMC1955440; doi:10.1186/1471-2458-7-155)

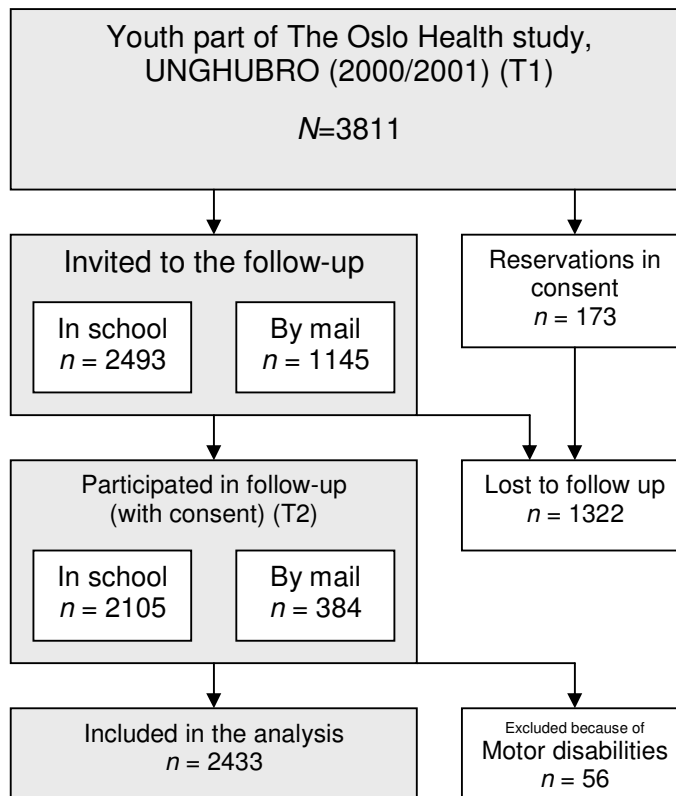

Supplement: Additional file 1 — Flowchart of the study and the participants at follow-up. [file 1471-2458-7-155-S1.pdf]

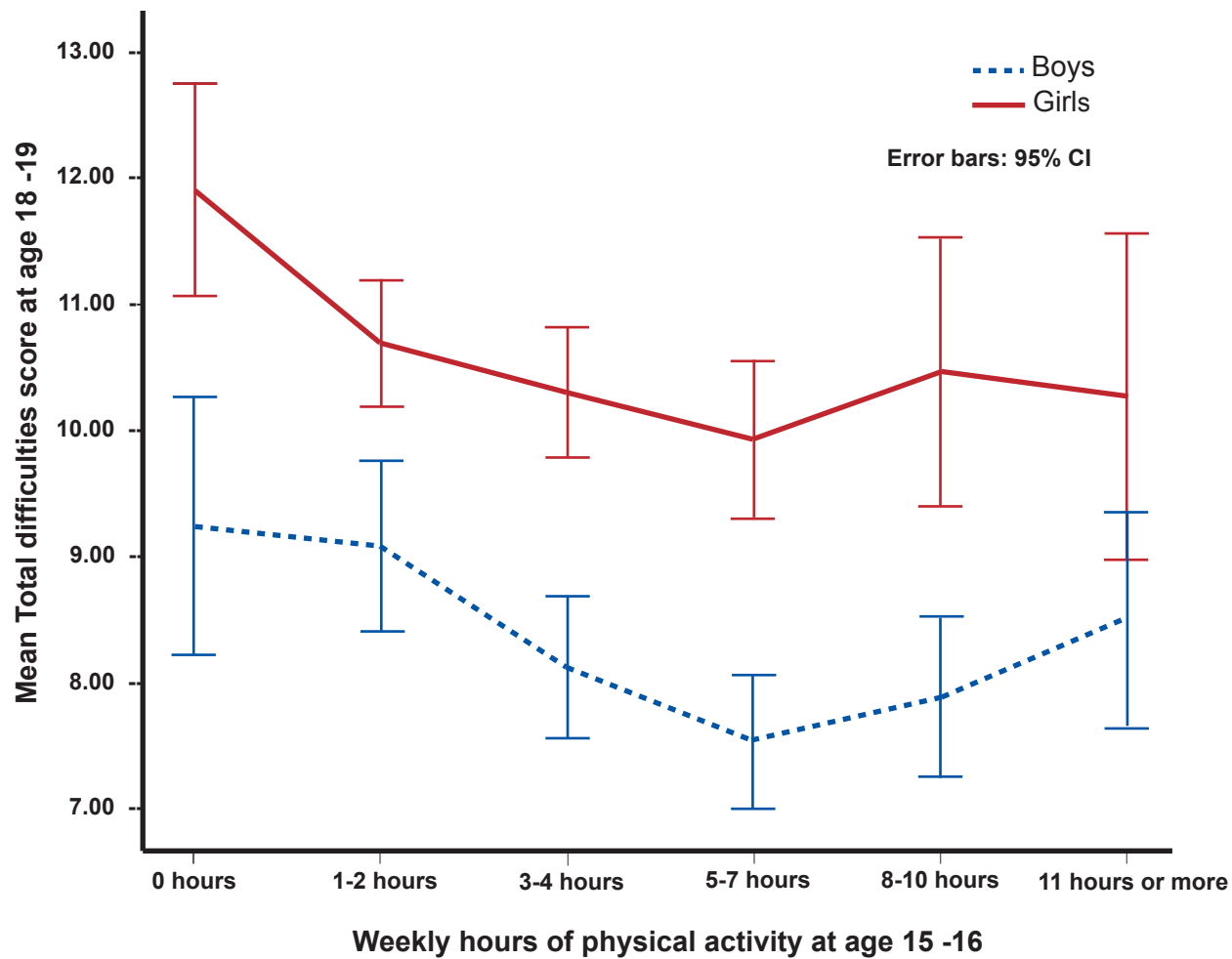

Supplement: Additional file 2 — Strengths and Difficulties Questionnaire Total difficulties score (follow up) according to weekly hours of physical activity (baseline) in boys and girls. [file 1471-2458-7-155-S2.pdf]
